# Preliminary assessment of sarcopenic obesity risk after sleeve gastrectomy using dual-modality imaging: a six-month MRI and DXA pilot study in women

**DOI:** 10.3389/fragi.2026.1913282

**Published:** 2026-07-31

**Authors:** Matej Pekar, Veronika Horka, Denisa Hyvlova, Radovan Jirik, Jaroslav Uchytil, Marketa Rygelova, Petr Kutac, Hana Tomaskova, Dominik Vilimek, Pavol Holeczy, Jan Maca, Marek Buzga

**Affiliations:** 1 Department of Physiology and Pathophysiology, Faculty of Medicine, University of Ostrava, Ostrava, Czechia; 2 Department of Physiology, Faculty of Medicine, Masaryk University, Brno, Czechia; 3 Complex Cardiovascular Center, Hospital AGEL Trinec-Podlesi, Trinec, Czechia; 4 Department of Human Movement Studies, Human Motion Diagnostic Centre, University of Ostrava, Ostrava, Czech Republic; 5 Institute of Scientific Instruments, Czech Academy of Sciences, Brno, Czechia; 6 Faculty of Electrical Engineering and Communication, Brno University of Technology, Brno, Czechia; 7 Institute of Epidemiology and Public Health, Faculty of Medicine, University of Ostrava, Ostrava, Czechia; 8 Department of Cybernetics and Biomedical Engineering, VSB-Technical University of Ostrava, Ostrava, Czechia; 9 Department of Surgery, Vitkovice Hospital AGEL, Ostrava, Czechia; 10 Department of Anesthesiology and Intensive Care Medicine, Faculty of Medicine, University Hospital Ostrava, University of Ostrava, Ostrava, Czechia; 11 Institute of Laboratory Diagnostics, University Hospital Ostrava, Ostrava, Czechia

**Keywords:** bariatric surgery, dual-energy x-ray absorptiometry, fat tissue, magnetic resonance imaging, muscles, sarcopenic obesity risk

## Abstract

**Introduction:**

Sarcopenic obesity (SO) is characterized by excess adiposity combined with skeletal muscle loss, but the absence of standardized diagnostic criteria makes interpretation of muscle tissue changes after bariatric surgery difficult, and different diagnostic indices can yield markedly different conclusions about the same patients.

**Methods:**

This preliminary analysis from the SarxOb trial (NCT04617392) compared several body-composition indices for detecting SO risk in eleven female patients undergoing laparoscopic sleeve gastrectomy, prospectively followed for six months using dual-modality imaging — magnetic resonance imaging (MRI) and dual-energy X-ray absorptiometry (DXA).

**Results:**

Lean body mass (LBM) decreased significantly from 56.3 ± 6.2 kg to 47.6 ± 6.0 kg (p < 0.001), while LBM as a percentage of total body weight increased from 50.4 ± 3.2% to 57.9 ± 4.3% (p = 0.003), reflecting a disproportionately greater loss of fat mass than lean mass. Appendicular skeletal muscle mass (ASM) decreased from 24.5 ± 3.3 kg to 20.1 ± 2.9 kg (p < 0.001). Critically, the choice of diagnostic index changed the apparent prevalence of SO risk entirely: by the ASM and ASM/height^2^ indices, no patient met criteria for SO risk at any timepoint, whereas by the weight-adjusted ASM/weight index recommended by the 2022 ESPEN/EASO consensus statement, ten of eleven patients met SO-risk criteria at baseline and at one month postoperatively, decreasing to four of eleven by six months. MRI-based quantification of the right lower limb corroborated the DXA findings, showing a parallel increase in the muscle-to-adipose tissue ratio.

**Conclusion:**

These results indicate that weight-adjusted indices detect a substantially higher burden of SO risk than traditional height-adjusted indices in this population, and that the choice of diagnostic index, not only the imaging modality used, is a major source of disagreement in reported SO risk prevalence after bariatric surgery. Given the small sample size inherent to this interim analysis of an ongoing trial, these findings should be considered exploratory and hypothesis-generating rather than confirmatory. As functional muscle assessment was not available in this interim report, these findings should be interpreted as SO risk by body-composition criteria rather than a complete EWGSOP2/ESPEN-EASO diagnosis.

## Introduction

Evidence from recent decades shows that bariatric surgery interventions are the only long-term effective approach achieving significant weight loss in patients with severe obesity (O'Brien et al., 2019). Treatment success is generally defined as achieving 50% Excess Weight Loss (EWL) or 20% Total Weight Loss (TWL) during the first year of treatment (Grover et al., 2019). Despite the undisputed benefit of weight reduction, these interventions also bring complications. One of the currently studied complications is the impact on skeletal muscle tissue. While the primary aim of bariatric interventions is absolute weight reduction and particularly decreased adipose tissue volume, this reduction is often accompanied by a decrease in skeletal muscle mass. Rapid weight reduction in the first months after surgical intervention represents a significant benefit; however, substantial muscle tissue reduction leads to long-term negative changes affecting both muscle metabolism and functionality (Seo et al., 2024; Gomez-Ambrosi et al., 2017). Decreased muscle mass in bariatric patients increases the risk of health complications, mainly of the cardiovascular system, worsens insulin resistance, and has been shown to increase the incidence of postoperative complications of gastrointestinal resection procedures, impair cognitive function, and impair healing of acute and chronic wounds (Tabesh et al., 2022; Haghighat et al., 2022; Nuijten et al., 2022; Van De Laar et al., 2018).

Recent data emphasize the importance of proactive monitoring and interventions focused on body composition changes and skeletal muscle health, particularly in cases of significant weight reductions exceeding 50% EWL or 20% TWL during the first year after surgery (Walowski et al., 2020).

In the last decade, attention has turned to the risk of sarcopenia, or more specifically, sarcopenic obesity (SO). The term “sarcopenia” was first defined by Rosenberg in 1988 as age-related skeletal muscle mass loss and weakness in older adults (Rosenberg, 1997). With accumulating evidence and research, the original definition expanded to include pathological muscle changes accompanying functional deterioration due to disease processes, such as obesity (Prado et al., 2012). This led to the recognition of a distinct subtype of sarcopenia known as SO (Prado et al., 2012). Consensus defines sarcopenic obesity as a condition characterized by low lean muscle mass in the presence of obesity. In this context, it is important to recognize that skeletal muscle is a significant endocrine-behaving tissue with a crucial role in the physiological metabolism of basic macronutrients, particularly glucose and glycogen (Hoffmann and Weigert, 2017; Batsis and Villareal, 2018). Despite significant progress in the diagnosis and pathophysiology of sarcopenia itself, the pathophysiology of sarcopenic obesity is not fully understood. However, obesity appears to be an independent factor affecting muscle tissue through adipose tissue (Hoffmann and Weigert, 2017). One of the most extensively studied mechanisms is the influence of adipokine secretion and pro-inflammatory cytokines by adipose tissue, which directly affect muscle catabolism of sugars and amino acids (Donini et al., 2022).

While SO is conceptually defined and various diagnostic technologies are available, the absence of standardized diagnostic criteria remains a significant challenge for clinical assessment (Ji et al., 2022). The aim of our study was to evaluate the occurrence of sarcopenic obesity risk in patients undergoing bariatric treatment, specifically sleeve gastrectomy, using advanced imaging methods and diagnostic criteria recommended by expert panels: the European Society for Clinical Nutrition and Metabolism (ESPEN), the European Association for the Study of Obesity (EASO), and the European Working Group on Sarcopenia in Older People (EWGSOP2) (Donini et al., 2022; Cruz-Jentoft et al., 2019). A second, central aim was to compare commonly used diagnostic indices for SO detection—derived from DXA and MRI—against one another within the same patients, since indices that disagree about which patients are at risk complicate both clinical decision-making and comparison of research outcomes across studies.

## Methods

The study was approved by the local Ethics Committee (Hospital AGEL Ostrava-Vitkovice), in accordance with the ethical standards of the Declaration of Helsinki and its subsequent amendments. Written informed consent was obtained from all participants for both study participation and publication of anonymized research outcomes. This analysis presents preliminary 6-month body-composition data from eleven female participants enrolled in the ongoing SarxOb trial (NCT04617392), an open-label randomized controlled trial whose complete design has been described in a separate protocol publication (Buzga et al., 2023). This secondary comparative analysis of pre-specified body-composition endpoints falls within the scope of the original ethics approval and registered protocol (Buzga et al., 2023); no additional ethics amendment was required. Feasibility outcomes from this same baseline-to-6-month cohort—including dual-modality (DXA/MRI) protocol completion rates, MRI segmentation algorithm performance, and a preliminary evaluation of the ASM/weight cut-off—have been reported separately (Buzga et al., 2025). The present analysis uses the same body-composition dataset but addresses a distinct question not covered in that report: a head-to-head comparison of ASM, ASM/height^2^, and ASM/weight as diagnostic indices within the same patients, to determine how much of the disagreement in reported sarcopenic obesity prevalence reflects index choice rather than imaging modality. Within the parent trial, participants are randomized to a supervised exercise intervention plus standard post-bariatric care (n = 5) or to standard care alone (n = 6); because this report focuses on body-composition and imaging methodology rather than intervention efficacy, and because arm sizes at this interim stage are too small to support a meaningful between-group comparison, all eleven participants are analyzed here as a single pooled cohort. Between-arm comparisons of intervention efficacy will be reported separately once the full trial cohort and 18-month follow-up are complete.

The study was conducted at the Obesity Research Center of the University of Ostrava and the Bariatric Surgery Center, Hospital AGEL Ostrava-Vitkovice. Inclusion criteria were age 18–65 years and BMI >40 kg/m^2^, or BMI >35 kg/m^2^ with comorbidities, following International Federation for the Surgery of Obesity and Metabolic Disorders (IFSO) criteria (Eisenberg et al., 2022). Sleeve gastrectomy was carried out using laparoscopic surgery as described in our previous study (Buzga et al., 2017). Exclusion criteria included prior major gastrointestinal surgery, diagnosis of gastric or duodenal ulcers, and gastrointestinal disease associated with a resorption disorder.

### Magnetic resonance imaging (MRI) and dual-energy X-Ray absorptiometry (DXA)

Body composition was assessed using DXA (Discovery A; Hologic, Waltham, MA, United States of America). The following parameters were monitored: fat mass (kg), fat (%), lean body mass (LBM, kg), and visceral adipose tissue (VAT, kg and cm^2^). One month before surgery and at one and 6 months after bariatric surgery, patients underwent MRI of the right lower limb with subsequent fat and muscle content quantification. All MRI data were acquired on a 1.5 T S Magnetom Sempra (Siemens, Erlangen, Germany) using a 6-channel body coil placed over the right leg. The imaging protocol consisted of T1w, T2w, and T1 VIBE Dixon sequences. A detailed description of MRI parameters can be found in the protocol article (Buzga et al., 2023).

### MRI data analysis and fat quantification

A preliminary algorithm for automatic segmentation and quantification of adipose and muscle tissue from MRI data was implemented in Python (version 3.10.12). The input for segmentation consisted of fat-only and water-only Dixon images of a manually selected slice from the center of the thigh, selected according to the anatomy of major muscles, vessels, and nerves. The algorithm output was the percentage of adipose and muscle tissue.

Before segmentation, phase-induced fat-water swaps were automatically detected and corrected. For this purpose, the manually selected slice was analyzed by detecting the largest object in both fat-only and water-only images using the OpenCV findContours function. A common bounding box covering the largest object in both images was determined, and the mean normalized signal intensity was computed within the central one-third region of the bounding box. Since skeletal muscle occupies the central part of the thigh and exhibits a higher signal intensity in the water-only image than in the fat-only image, the images were considered swapped if the mean normalized intensity was higher in the fat-only image, and were subsequently exchanged.

The first step in the segmentation process involved applying Otsu thresholding independently to the fat-only and water-only images, followed by removing connected components smaller than 50 pixels. This produced the initial fat and water segmentations. The binarized fat-only image was then used to identify the full-leg segment in the slice, which was essential for subsequent tissue percentage quantification. Full-leg segmentation was performed by selecting the largest connected component in the binarized fat-only image, filtering it using morphological closing with a 13 × 13 kernel, followed by binary hole filling. The full-leg segment contained, apart from the sought adipose and muscle tissue, other tissues present in the leg (e.g., bone, vessels, or nerves).

Subsequently, rough fat and water segmentations were smoothed using morphological closing with a 3 × 3 kernel. The contralateral leg, which was partially captured in both images, along with fascia present in the water-only image, were suppressed from the segmentations using the full-leg segment as a mask and applying morphological opening with a 3 × 3 kernel. The resulting fat-only and water-only masks represented the final adipose tissue and muscle segmentations, respectively. Finally, in the rare cases where pixel assignment conflicts occurred between tissue categories, the affected pixels were assigned to muscle tissue.

Tissue percentages were calculated from the ratio of the number of pixels corresponding to a particular tissue type to the total number of pixels in the full-leg segment. Analyses were conducted for baseline measurements and measurements taken at 1 and 6 months. The algorithm’s performance was evaluated using the Dice coefficient. Ground truth data were segmented by an expert radiologist. The entire leg was annotated, excluding slices affected by artifacts, resulting in a total of 413 ground-truth slices. The mean Dice scores were then computed across all slices and datasets for the two classes (muscle and adipose tissue), using the formula: Dice = 2 × TP/(2 × TP + FP + FN), where TP, FP, and FN represent true positives, false positives, and false negatives, respectively.

### Definitions of obesity and sarcopenic obesity risk

Obesity was defined as BMI >30 kg/m^2^. Three indices of muscle mass were calculated from DXA data: appendicular skeletal muscle mass (ASM, kg), defined as the sum of lean mass in the arms and legs (Donini et al., 2022; Cruz-Jentoft et al., 2019); ASM divided by height squared (ASM/height^2^, kg/m^2^) (Tagliafico et al., 2022); and ASM divided by body weight (ASM/weight), adjusted per current sarcopenic obesity recommendations (Poggiogalle et al., 2016). The cut-offs applied were ASM <15 kg for women (Donini et al., 2022; Cruz-Jentoft et al., 2019), ASM/height^2^ < 5.67 kg/m^2^ (Tagliafico et al., 2022), and ASM/weight <0.235 (Poggiogalle et al., 2016). We use the term “SO risk” rather than “SO diagnosis” throughout this report because a complete EWGSOP2/ESPEN-EASO diagnosis additionally requires a functional muscle assessment (e.g., handgrip strength), which was not available at these timepoints; this point is discussed further in the Limitations section. Data obtained from DXA were used for all index calculations.

### Statistical evaluation

Standard methods of descriptive statistics (means, standard deviations, and medians) were used for data presentation. Within-subject changes from baseline to 1 month and from baseline to 6 months were evaluated using paired t-tests or Wilcoxon signed-rank tests, depending on the distribution of the data. All analyses were performed in Stata (version 17) at a 5% significance level. Because the underlying body-composition measures analyzed here were pre-specified secondary outcomes of the registered SarxOb trial protocol (Buzga et al., 2023), and the index-comparison analysis presented in this report is a planned secondary synthesis of those measures rather than an exploratory subgroup analysis, no correction for multiple comparisons was applied. Normality of each variable’s within-subject differences was assessed using the Shapiro-Wilk test (α = 0.05); the paired t-test was used where normality was not rejected, and the Wilcoxon signed-rank test otherwise. Tables 2, 3 indicate, by footnote, which test was applied to each variable. For every within-subject comparison, Tables 2, 3 additionally report the 95% confidence interval for the mean paired difference and Cohen’s dz as a standardized paired-samples effect size with its own 95% CI, to aid interpretation of these estimates in this small pilot sample.

## Results

### Patient group description at baseline

Eleven women were enrolled in this analysis. All patients underwent laparoscopic sleeve gastrectomy. The mean age at the time of the procedure was 41.2 ± 7.9 years. All participants were Caucasian. Baseline characteristics of the study population are detailed in Table 1.

**TABLE 1:** Baseline characteristics of the patients included in the study.

Variable	Value
N	11
Age (years), mean ± SD	41.2 ± 7.91
Height (cm)	165.5 ± 5.64
BMI (kg/m^2^)	41.0 ± 4.98
HbA1c (mmol/mol), mean ± SD	37.10 ± 4.38
HbA1c (% DCCT), mean ± SD	5.53 ± 0.38
Type 2 diabetes (%)	0
Arterial hypertension (%)	9.1

Baseline demographic and clinical characteristics of the study population (N = 11). Data are presented as mean ± standard deviation (SD) or percentages. BMI, body mass index; HbA1c = Glycated Hemoglobin; DCCT, diabetes control and complications trial.

### Weight reduction and DXA body composition

Body weight decreased significantly from baseline to 6 months post-procedure (112.4 ± 15.6 vs. 82.9 ± 14.5 kg, p < 0.001), an average loss of 29.4 kg. BMI also decreased significantly (41.0 ± 4.98 vs. 30.3 ± 5.3 kg/m^2^, p < 0.001), an average reduction of 10.6 kg/m^2^. At 6 months, total weight loss (TWL) was 26.3% ± 5.6%, and excess weight loss (EWL) was 73.7% ± 5.6%.

DXA analysis revealed a significant decrease in both adipose tissue mass (56.0 ± 10.3 vs. 35.4 ± 9.2 kg, p < 0.001) and adipose tissue percentage (50.2% ± 3.2% vs. 42.8% ± 4.3%, p = 0.003). Visceral adipose tissue (VAT) decreased significantly in both mass (858.9 ± 133.1 vs. 615.1 ± 213.9 g, p < 0.001) and area (189.7 ± 49.1 vs. 136.8 ± 57.5 cm^2^, p = 0.004). LBM decreased significantly in absolute terms (56.3 ± 6.2 vs. 47.6 ± 6.0 kg, p < 0.001), while LBM as a percentage of total body weight increased from 50.4% ± 3.2% to 57.9% ± 4.3% (p = 0.003) over the same period—a pattern consistent with proportionally greater loss of fat mass than lean mass. In contrast to absolute LBM, this relative shift toward preserved lean mass was not yet apparent at 1 month: LBM (%) was essentially unchanged from baseline at this timepoint (48.9% ± 3.2%, p = 0.424) and only increased significantly by 6 months. All body composition data are presented in Table 2.

**TABLE 2:** Body composition changes during 6-month follow-up after sleeve gastrectomy.

Variable	Baseline	Month 1	[95% CI] P-value (test)	Cohen’s dz [95% CI]	Month 6	[95% CI] P-value (test)	Cohen’s dz [95% CI]
Weight (kg)	112.4 ± 15.6	101.9 ± 14.9	[5.8, 16.6] <0.001 (wilcoxon)	4.01 [2.17, 5.83]	82.9 ± 14.5	[15.2, 43.8] <0.001 (paired t-test)	4.32 [2.34, 6.31]
DXA LBM (kg)	56.3 ± 6.2	49.2 ± 5.8	[3.1, 8.9] <0.001 (paired t-test)	2.53 [1.28, 3.76]	47.6 ± 6.0	[4.5, 12.9] <0.001 (paired t-test)	2.86 [1.47, 4.24]
DXA LBM (%)	50.4 ± 3.2	48.9 ± 3.2	[−2.5, 5.5] 0.424 (paired t-test)	0.20 [-0.40, 0.80]	57.9 ± 4.3	[−11.8, −3.2] 0.003 (paired t-test)	−3.50 [−5.14, −1.85]
DXA fat (kg)	56.0 ± 10.3	53.3 ± 9.7	[2.6, 7.6] <0.001 (paired t-test)	2.11 [1.01, 3.17]	35.4 ± 9.2	[10.6, 30.6] <0.001 (paired t-test)	4.55 [2.47, 6.63]
DXA fat (%)	50.2 ± 3.2	51.0 ± 3.2	[−1.7, −0.9] 0.003 (paired t-test)	−0.20 [-0.80, 0.40]	42.8 ± 4.3	[3.9, 11.1] 0.003 (paired t-test)	3.49 [1.85, 5.13]
DXA VAT (g)	858.9 ± 133.1	848.0 ± 185.9	[14.6, 42.0] <0.001 (paired t-test)	0.42 [0.21, 1.03]	615.1 ± 213.9	[125.4, 362.2] <0.001 (paired t-test)	1.94 [0.90, 2.97]
DXA VAT (cm^2^)	189.7 ± 49.1	182.4 ± 53.4	[−0.4, 15.0] 0.062 (paired t-test)	0.42 [-0.21, 1.03]	136.8 ± 57.5	[21.2, 84.6] 0.004 (paired t-test)	1.94 [0.90, 2.97]

Changes in body composition parameters measured by DXA, at baseline, 1 month, and 6 months post-surgery. Data are presented as mean ± standard deviation with 95% confidence interval. P-values compare changes from baseline; the statistical test used for each comparison is shown in parentheses. LBM, lean body mass; VAT, visceral adipose tissue; kg = kilograms; g = grams; cm^2^ = square centimeters. VAT (g) = Visceral Adipose Tissue Mass; VAT (cm^2^) = Visceral Adipose Tissue Area.

### MRI data segmentation algorithm performance

**FIGURE 1:**
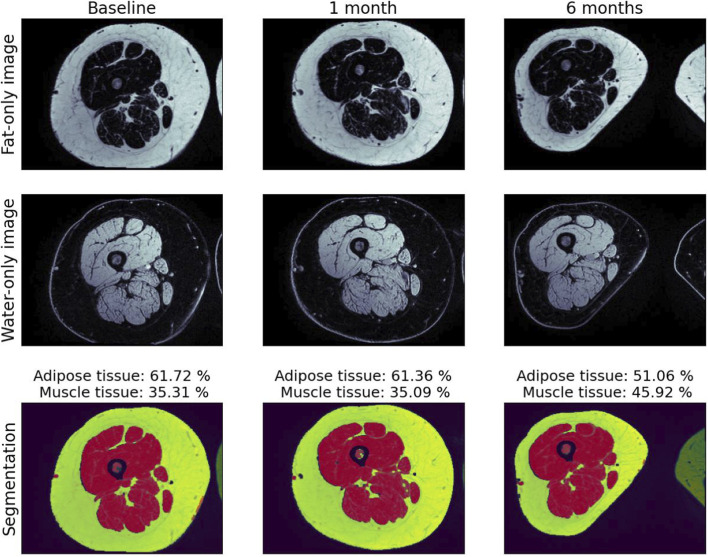
Mri analysis of right lower limb tissue composition at baseline, 1 month, and 6 Months post-surgery. Legend: Representative MRI images showing changes in adipose and muscle tissue composition of the right lower limb. Top row: Fat-only images. Middle row: Water-only images. Bottom row: Segmentation results with adipose tissue (yellow) and muscle tissue (red). Quantitative measurements of tissue proportions are shown below for each time point. The images demonstrate progressive changes in tissue composition over the 6-month follow-up period.

The mean Dice coefficient achieved by our algorithm was 0.95 ± 0.01 for muscle tissue and 0.95 ± 0.03 for adipose tissue. Figure 1 displays examples of MRI data and segmentations, showing fat-only images (row 1), water-only images (row 2), and resulting segmentation (row 3; yellow–adipose tissue, red–muscle tissue) for all three acquisitions.

The preliminary approach successfully segmented adipose and muscle tissues, and the algorithm effectively excluded vessels and other extraneous structures. However, bone marrow was misclassified as muscle tissue in the example segmentations in Figure 1 in all three acquisitions. Careful inspection of segmentation results across all patients showed that while the preliminary algorithm was consistent within single-patient measurements, it varied between patients (classifying bone marrow either as muscle, adipose tissue, or correctly excluding it). While we acknowledge this inter-patient bias and plan to refine the algorithm in future work, the estimated metrics remained consistent for intra-patient acquisitions, ensuring valid temporal comparisons.

### Quantitative analysis of muscle and adipose tissue from MRI data

**FIGURE 2:**
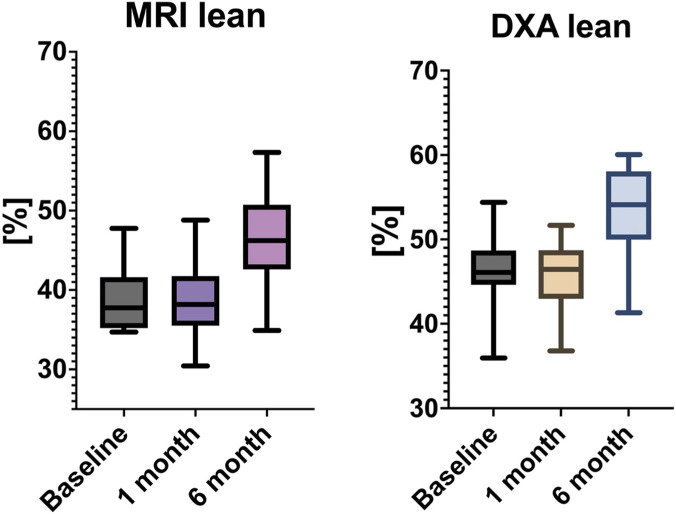
Comparison of lean tissue assessment between MRI and DXA analysis. Legend: Box plots comparing lean tissue percentage measurements using MRI (left panel) and DXA (right panel) at baseline, 1 month, and 6 months post-surgery. The plots demonstrate the temporal evolution of lean tissue composition as measured by both imaging modalities. Boxes represent interquartile range with median line; whiskers show minimum and maximum values.

**FIGURE 3:**
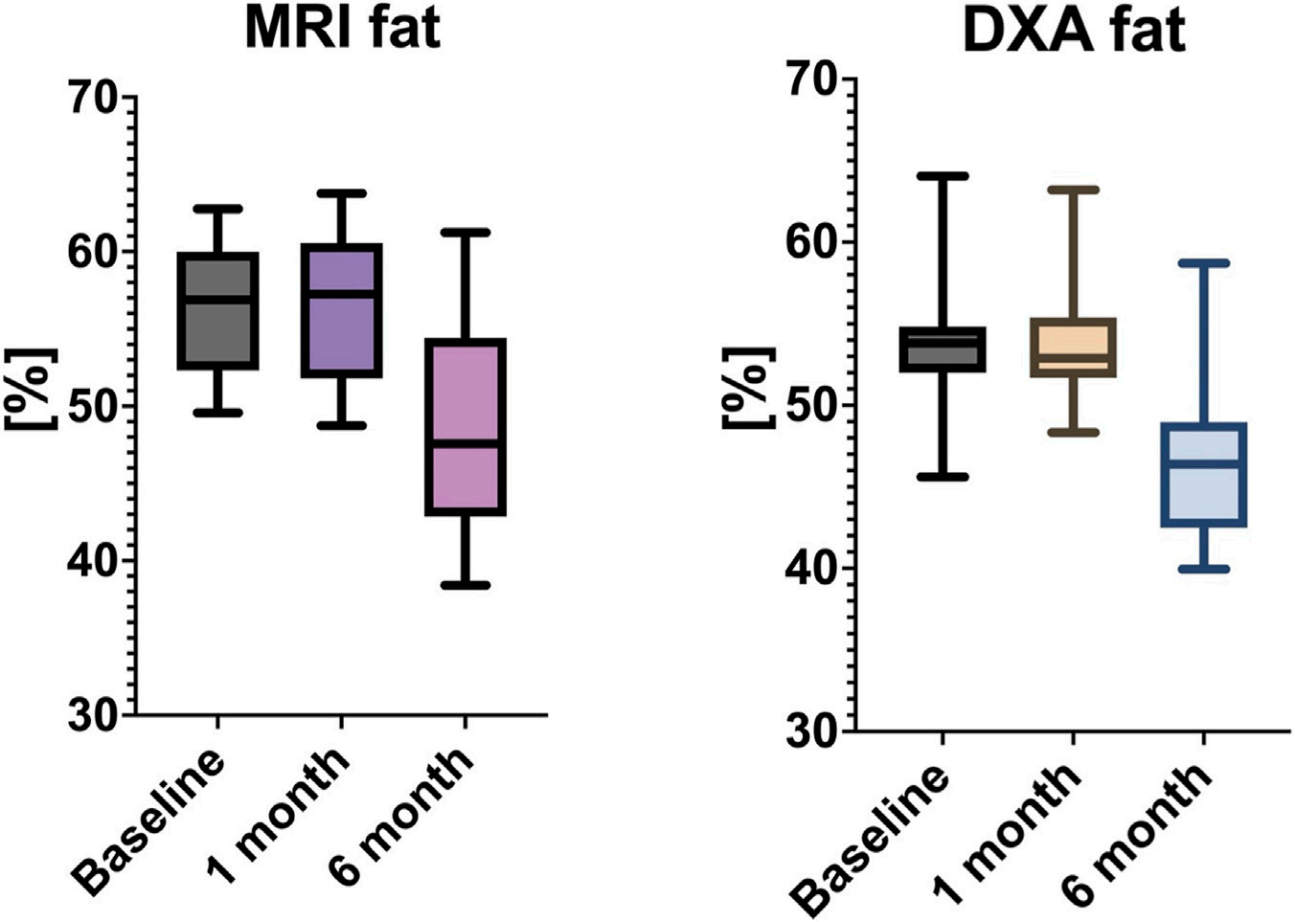
Comparison of adipose tissue percentage between MRI and DXA analysis. Legend: Box plots comparing adipose tissue percentage measurements using MRI (left panel) and DXA (right panel) analysis at baseline, 1 month, and 6 months post-surgery. Both imaging modalities show consistent trends in adipose tissue reduction over the follow-up period. Boxes represent interquartile range with median line; whiskers extend to minimum and maximum values.

**FIGURE 4:**
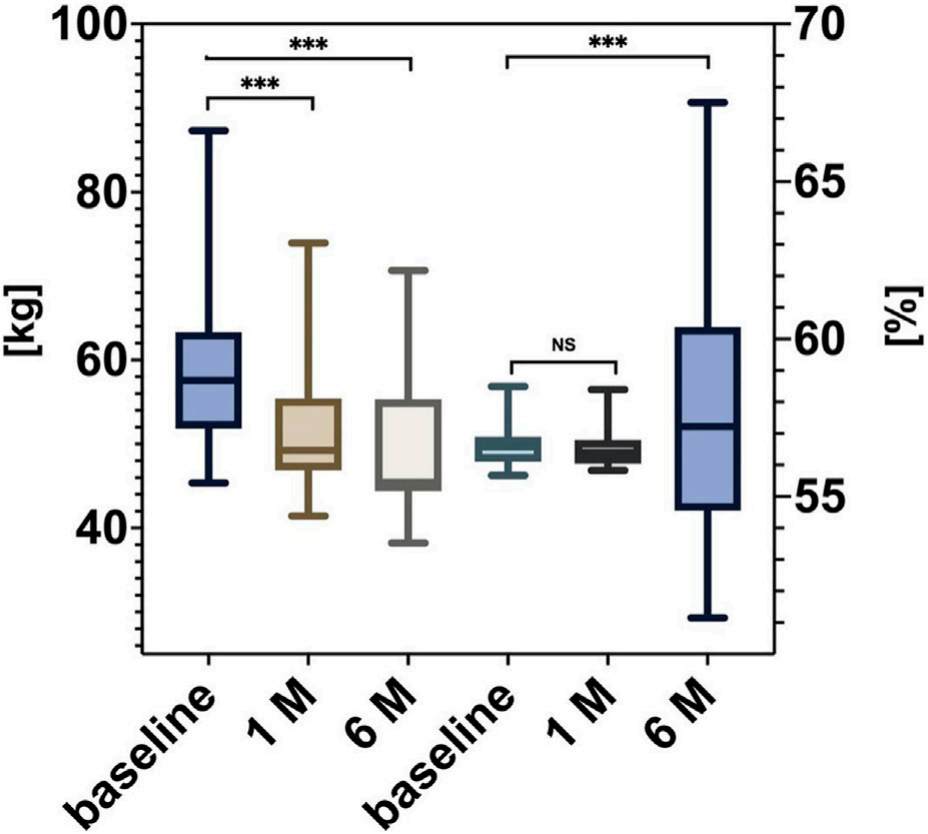
Dual-energy X-Ray absorptiometry assessment of lean body mass changes. Legend: Box plot demonstrating post-bariatric surgery changes in lean body mass. Left y-axis and first three boxes show absolute lean mass values (kg), revealing a significant decrease from baseline through 6 months (***p < 0.001). Right y-axis and last three boxes represent lean mass as a percentage of total body mass, showing significant improvement over the same period (***p < 0.001). This pattern demonstrates that despite the significant decrease in absolute lean body mass, patients exhibited an improved body composition profile with increased relative lean mass percentage, reflecting a more favorable tissue distribution following bariatric surgery. Boxes represent interquartile range with median line; whiskers extend to minimum and maximum values. Time points: baseline, 1 month (1 M), and 6 months (6 M) post-surgery.

MRI analysis revealed notable changes in the ratio of adipose tissue to skeletal muscle in the cross-sectional area of the right lower limb, favoring muscle tissue. The proportion of adipose tissue decreased from a baseline of 58.0% ± 5.0% to 49.2% ± 6.1% at 6 months postoperatively (p < 0.001). Conversely, muscle tissue percentage in the right lower limb increased from 38.7% ± 5.2% at baseline to 47.3% ± 5.8% at 6 months postoperatively (p < 0.001). These changes are illustrated in Figures 2–4.

### Sarcopenic obesity risk based on diagnostic indices

ASM decreased significantly from 24.5 ± 3.3 kg at baseline to 20.1 ± 2.9 kg at 6 months (p < 0.001), and ASM/height^2^ also declined significantly over the same period (p < 0.001; Table 3). According to established cut-off values, no patient met criteria for sarcopenic obesity risk by either the ASM or ASM/height^2^ index at any timepoint. In marked contrast, when ASM was adjusted for body weight per current EWGSOP2/ESPEN-EASO recommendations (Prado et al., 2012; Ji et al., 2022), ten of eleven subjects met the threshold for sarcopenic obesity risk by the ASM/weight index at baseline and at 1 month postoperatively. By 6 months, this had improved substantially, with only four of eleven patients still meeting the ASM/weight criterion. Complete index data are presented in Table 3.

**TABLE 3:** Diagnostic indices for sarcopenic obesity risk assessment over 6-month follow-up.

Variable	Baseline	Month 1	[95% CI] P-value (test)	Cohen’s dz [95% CI]	Month 6	[95% CI] P-value (test)	Cohen’s dz [95% CI]
ASM (kg)	24.5 ± 3.3	21.9 ± 3.0	[1.3, 3.9] <0.001 (paired t-test)	3.35 [1.78, 4.89]	20.1 ± 2.9	[2.3, 6.5] <0.001 (paired t-test)	3.50 [1.87, 5.12]
ASM/height^2^	8.9 ± 1.0	8.0 ± 0.9	[0.5, 1.3] <0.001 (paired t-test)	3.89 [2.10, 5.65]	7.3 ± 1.0	[0.8, 2.4] <0.001 (paired t-test)	3.99 [2.16, 5.80]
ASM/weight	0.219 ± 0.01	0.212 ± 0.01	[−0.008, 0.022] 0.318 (paired t-test)	0.32 [-0.30, 0.92]	0.245 ± 0.02	[−0.039, −0.013] <0.001 (paired t-test)	−3.25 [-4.77, −1.72]

Changes in sarcopenic obesity risk diagnostic indices at baseline, 1 month, and 6 months post-surgery. Data are presented as mean ± standard deviation with 95% confidence interval. ASM, Appendicular Skeletal Muscle Mass. P-values compare changes from baseline; the statistical test used for each comparison is shown in parentheses. Cut-offs: ASM <15 kg, ASM/height^2^ < 5.67 kg/m^2^, ASM/weight <0.235.

## Discussion

This study evaluated body-composition changes and sarcopenic obesity (SO) risk during the first 6 months after laparoscopic sleeve gastrectomy in middle-aged women, using dual-modality imaging (DXA and MRI) to compare how different diagnostic indices classify the same patients. One of the most notable findings is that index choice, not just measurement modality, appears to be associated with whether SO risk is detected at all in this population: the standard ASM and ASM/height^2^ indices identified no patient as at risk at any timepoint, while the weight-adjusted ASM/weight index—the parameter recommended by the 2022 ESPEN/EASO consensus statement for patients with obesity (Donini et al., 2022) — identified ten of eleven patients as at risk before surgery, decreasing to four of eleven by 6 months. This magnitude of disagreement between indices, in the same patients measured with the same instrument, illustrates why standardized diagnostic criteria for SO remain an unresolved problem in this population, and suggests that reported SO prevalence in the bariatric literature may depend as much on which index a study selects as on the underlying biology.

This pattern is consistent with the broader paradox observed in absolute and relative measures of lean tissue. While absolute LBM decreased as expected with major weight loss (56.3 ± 6.2 to 47.6 ± 6.0 kg, p < 0.001), the proportion of LBM relative to total body weight increased significantly (50.4% ± 3.2% to 57.9% ± 4.3%, p = 0.003), because fat mass was lost disproportionately faster than lean mass. This favorable shift was not immediate: as noted in Results, LBM (%) and ASM/weight were both essentially unchanged from baseline at 1 month (p = 0.424 and p = 0.318, respectively) and only diverged favorably by 6 months (Tables 2, 3), suggesting that the relative preservation of muscle mass becomes detectable only several months into recovery—a pattern with potential implications for the timing of postoperative nutritional or exercise support. Our MRI analysis of the right lower limb corroborated the overall pattern, showing a parallel increase in the muscle-to-adipose tissue ratio within the imaged cross-section. Taken together, these findings indicate that absolute muscle mass loss after bariatric surgery, considered on its own, can be a misleading metric of SO risk; relative, weight-adjusted measures appear to track the postoperative trajectory more informatively.

This convergence between DXA-derived and MRI-derived measures strengthens confidence in the underlying biological signal, since the two modalities use different physical principles to quantify tissue composition. It also illustrates the value of a dual-modality approach for interpreting SO risk after bariatric surgery: where DXA alone might suggest a straightforwardly negative effect of surgery on muscle mass, the addition of relative and imaging-based measures reveals a more complete picture, in which the muscle-to-fat balance improves over the same postoperative period even as absolute muscle mass declines—though, because this interim cohort pools a supervised-exercise arm with standard care, this improvement cannot yet be attributed to surgery alone.

Recent studies and expert panel consensus show that severe obesity strongly correlates with chronic metabolic diseases such as type 2 diabetes mellitus (DM2T), hypertension, and dyslipidemia (Powell-Wiley et al., 2021; Schauer et al., 2017). Combined with reduced locomotion, which is part of the lifestyle in obese patients, these factors lead to SO development. Some research suggests that SO risk exists even in patients undergoing treatment for severe obesity through bariatric surgery (Sjostrom et al., 2004).

In recent years, several recommendations for SO assessment have been published, both in terms of recommended methods and cut-off values (Donini et al., 2022). Unfortunately, we currently have multiple definitions and lack consensus regarding ideal body composition assessment methods. These discrepancies complicate both SO risk assessment and comparison of research outcomes that could be evaluated in meta-analyses.

In our study, DXA and its derived sarcopenic indices were primarily used for SO risk assessment. Interestingly, using EWGSOP2 criteria with the ASM and ASM/height^2^ indices, none of the patients met SO-risk criteria. However, using ASM/weight, which some authorities recommend, most patients met sarcopenic obesity risk criteria before surgery. This risk decreased in most women in our cohort 6 months post-surgery. Similar results were reported by Poggiogalle et al. (Poggiogalle et al., 2016) in a cohort of 727 obese patients, where no patients were at SO risk using standard indices, but when evaluated by ASM/weight, the original prevalence increased from 1% to 50.1%. A similar pattern of high SO-risk values in women undergoing bariatric surgery was recently reported by Rodrigues et al., in 2024 in a cohort of 140 women (Rodrigues et al., 2024). This raises the question of whether current standard indices used by European and American professional societies are sufficient, and whether they need adjustment for body weight, especially in highly obese patients.

These studies and our data contrast with other studies on SO prevalence in patients treated with bariatric methods. Recent data show prevalence between 4.4% and 30% (Gonzalez Arnaiz et al., 2024). However, the problem extends beyond inconsistent methodology and criteria to the diagnostic methods used. Recent recommendations prefer CT or MRI, as these are the only methods enabling direct skeletal muscle tissue assessment. DXA and bioelectrical impedance analysis (BIA) are considered secondary examinations (Heymsfield et al., 2015; Schautz et al., 2012). Unlike CT or MRI, DXA can only examine soft tissue summarily and separate bone and fat mass. Consequently, only first-choice methods (CT or MRI) provide a more detailed picture. Some authors consider BIA methods of limited relevance to current recommendations (Sizoo et al., 2021), though some studies derive SO prevalence from BIA data (Crispim Carvalho et al., 2023). For example, Molero et al. (Molero et al., 2020) found 16.4% SO prevalence in a retrospective sample of 1,370 patients. A significant problem in some studies is the absence of cut-off values for SO assessment, even when using the ASM/weight index (Crispim Carvalho et al., 2023).

Success in treating severe obesity, both conservative and surgical, is primarily evaluated through weight reduction effectiveness. In our and similar studies, there was a concurrent reduction in absolute skeletal muscle mass. However, the question remains whether this assessment alone is sufficiently informative (Matos et al., 2020). In our study, absolute skeletal muscle mass decreased during follow-up. Nevertheless, when ASM/weight was considered and LBM percentages recalculated, there was, paradoxically, an increase in relative muscle mass. These data align with recent studies that noted an improved fat-to-muscle ratio in groups losing more than 50% excess weight, despite decreased absolute muscle mass (Crispim Carvalho et al., 2023; Sivakumar et al., 2022).

The selection of ASM/weight as our primary index for sarcopenic obesity risk assessment was based on current expert consensus recommendations. According to the 2022 ESPEN/EASO Consensus Statement, the diagnosis of sarcopenia in obese individuals requires relative reduction in skeletal muscle mass adjusted for body weight, with ASM/weight identified as the most suitable parameter when DXA is available (Donini et al., 2022).

This recommendation stems from observations that relative muscle mass reduction in the presence of high total body weight and fat mass can have significant clinical and functional impacts even without absolute muscle mass reduction (Sizoo et al., 2021). Height-squared correction, while common in sarcopenia assessment, correlates with BMI and may underestimate sarcopenia risk in individuals with obesity (Kim et al., 2016).

Studies show that patients can present with low relative muscle mass alongside excessive fat mass without low absolute muscle mass, resulting in adverse clinical consequences typical of sarcopenic obesity (Chen et al., 2016). Our approach aligns with these considerations, especially relevant in severe obesity where traditional indices may fail to identify sarcopenia risk.

The assessment of ASM indices with weight correction in bariatric patients remains limited in the literature, with Vieira et al. (Vieira et al., 2022) providing one of the few comprehensive evaluations. Our findings, in particular the magnitude of disagreement between indices observed within the same patients, contribute concrete data to this ongoing discussion regarding SO risk assessment in severely obese populations undergoing bariatric procedures.

MRI is increasingly considered a reference method for evaluating skeletal muscle changes directly, in contrast to DXA’s reliance on soft-tissue subtraction. In our study, similar to DXA, we observed an improved fat-to-muscle ratio favoring skeletal muscle using Dixon sequences, a finding that demonstrates the potential value of weight-adjusted calculations or more precise methods like MRI in future studies and clinical evaluations (Tagliafico et al., 2022; Matos et al., 2020; Ruthes et al., 2022). It is important to note, however, that validated MRI-specific cut-off values for diagnosing SO in bariatric or obese populations do not yet exist; the cut-offs applied in this study were developed for DXA-derived measures, and not for our MRI-based thigh segmentation. MRI’s contribution here is therefore best understood as a complementary, hypothesis-generating comparison against DXA-based indices, rather than as an independently validated diagnostic tool for SO in this population. We see this as a methodological strength rather than a weakness, since it identifies a concrete, well-defined target for future validation work using imaging-based muscle quantification. We agree with the ESPEN and EASO Consensus Statement conclusions recommending ASM/weight as the most suitable parameter when DXA is available (Donini et al., 2022).

Beyond corroborating DXA-derived trends, dual-modality imaging in this cohort points to several ways MRI could add unique diagnostic value in future work. Because our Dixon acquisitions separate fat and water signal voxel-by-voxel, the same images could, in principle, be used to quantify intramuscular and inter-muscular fat infiltration (myosteatosis) within the segmented thigh musculature (Chaudry et al., 2021; Grimm et al., 2018) — a marker of muscle quality that DXA cannot capture, since DXA reports only a single aggregate lean-mass value per region without distinguishing muscle quality from muscle quantity (Correa-de-Araujo et al., 2020). MRI is also independent of the fixed hydration constant that DXA’s lean-mass algorithm assumes (Wang et al., 1999; Pietrobelli et al., 1998); this may be particularly relevant during the early postoperative period, when fluid shifts are most pronounced and where, as noted above, the relative preservation of lean mass was not yet detectable by DXA at 1 month. Finally, unlike DXA, MRI involves no ionizing radiation (Seabolt et al., 2015), an advantage for a population that may benefit from repeated body-composition surveillance over many years. Realizing this potential will require the segmentation and validation work outlined in our Limitations section; we present it here as a rationale for pursuing MRI-based indices in future, larger-cohort analyses within the SarxOb trial, rather than as a capability already demonstrated in the present dataset.

Along with weight reduction, there was a significant reduction in both total and visceral adipose tissue. Adipose tissue, especially visceral, is a highly active endocrine organ that, particularly in severe obesity, is a source of inflammatory adipokines and cytokines (Hruska et al., 2022; Janochova et al., 2019). This condition, characterized by chronic inflammation, worsens various comorbidities such as DM2T, hypertension, atherosclerosis, and other serious diseases. Numerous studies have demonstrated a direct connection between VAT reduction and cardiovascular disease (Powell-Wiley et al., 2021). According to National Health and Nutrition Examination Survey data, people in the American population with central obesity had a higher cardiovascular mortality risk compared to those with the same BMI but without central adiposity (Coutinho et al., 2013). VAT is a highly metabolically active tissue that affects muscle tissue (Grimm et al., 2018). Some studies suggest that VAT volume reduction may lead to increased LBM (Cruz et al., 2011). The decrease in adipose tissue volume, along with an improved skeletal muscle profile, is likely one of the benefits that bariatric surgery has on DM2T and cardiovascular disease risk (Garneau and Aguer, 2019).

This report and our previously published feasibility analysis (Buzga et al., 2025) share the same baseline-to-6-month SarxOb cohort (n = 11) and dual-modality dataset, but answer different questions. The earlier report’s primary endpoints were protocol feasibility—completion rate, segmentation precision, tolerance—with ASM/weight prevalence reported only as a preliminary secondary outcome at baseline and 6 months. Having established feasibility, the present analysis asks how much the choice of diagnostic index, rather than imaging modality, accounts for disagreement in SO-risk classification. We extend the earlier analysis by: I. testing three indices against their cut-offs with paired significance testing at baseline, 1 month, and 6 months rather than two indices at two timepoints; II. showing that the 1-month timepoint—not previously analyzed statistically—is where the indices diverge most (SO-risk prevalence unchanged at 10/11 before falling to 4/11 b y 6 months); and III. using the MRI-derived muscle-to-adipose ratio comparatively, to test convergence across modalities, rather than correlating MRI against DXA as a validation exercise.

### Study strengths

This study offers several contributions to the field. Using ESPEN/EASO criteria, it is among the few investigations evaluating SO risk in middle-aged women after bariatric surgery, an understudied population. DXA was used as the primary body-composition assessment tool, in line with ESPEN/EASO recommendations given its accuracy relative to bioelectrical impedance analysis. Most notably, longitudinal MRI evaluation of skeletal muscle tissue was incorporated alongside DXA, allowing direct visualization and quantification of muscle tissue rather than DXA’s indirect soft-tissue assessment—and, as shown here, allowing diagnostic indices to be compared directly against an imaging-based reference within the same patients.

### Study limitations

Several limitations must be considered when interpreting these findings. First, and most importantly, this interim analysis assessed only body composition; no functional muscle assessment (e.g., handgrip strength, gait speed) was available at these timepoints. Because current EWGSOP2 and ESPEN/EASO frameworks require a functional criterion alongside reduced muscle mass to confirm a diagnosis of sarcopenia or sarcopenic obesity, the findings reported here should be interpreted as SO risk by body-composition criteria rather than a complete diagnosis; muscle strength and physical performance testing are planned as part of the full SarxOb protocol and will be reported separately. Second, this analysis proceeded directly to body-composition-based risk stratification without a preceding population-level screening step (e.g., the SARC-F questionnaire); because all participants had already met IFSO criteria for bariatric surgery, representing a population with confirmed severe obesity rather than an undifferentiated general population, we judged a screening step intended for case-finding to be less informative in this context than direct diagnostic assessment, though we acknowledge this departs from the stepwise screening-to-diagnosis structure recommended by ESPEN/EASO. Third, the small sample size (n = 11) limits statistical power and the generalizability of these findings; this report presents preliminary 6-month data from the ongoing SarxOb trial (NCT04617392), with complete 18-month outcomes, intervention-arm comparisons, and a larger combined cohort still pending. Fourth, this interim analysis pools participants from both arms of the underlying SarxOb trial—a supervised exercise intervention plus standard post-bariatric care (n = 5) and standard care alone (n = 6) — because between-arm comparisons are underpowered at this stage; as a structured exercise program is itself known to influence muscle preservation, the relative improvement in muscle-related indices reported here should not be attributed to bariatric surgery alone, and arm-specific comparisons will be reported once the full trial cohort is available. Fifth, this analysis was restricted to female participants, which limits generalizability to the broader bariatric surgery population; menopausal or climacteric status was not systematically recorded, which may be a relevant source of unmeasured variability in muscle and fat metabolism. In addition, all participants were of Caucasian ethnicity; given known ethnicity-specific differences in body composition and sarcopenia-related cut-off values (e.g., Asian Working Group for Sarcopenia criteria differ from the European consensus values applied here (Chen et al., 2016)), generalizability to other ethnic groups should be considered limited. Sixth, our MRI segmentation approach had specific technical limitations: the mean Dice coefficient achieved by our algorithm was 0.95 ± 0.01 for muscle tissue and 0.95 ± 0.03 for adipose tissue; for comparison, Yang et al. (Yang et al., 2016) reported Dice scores of 0.97 ± 0.01 and 0.96 ± 0.03 for muscle and subcutaneous adipose tissue, respectively, using an automated machine-learning approach, so our results are only marginally lower, likely reflecting our approach’s lack of separation between bone and inter-muscular adipose tissue. Bone marrow was consistently misclassified as muscle or adipose tissue in some patients, which likely overestimated absolute tissue volumes at all timepoints, and this misclassification varied between patients in a way that could bias group-level comparisons; because misclassification was consistent within each patient across timepoints, however, longitudinal within-subject comparisons should remain valid. Finally, as noted above, validated MRI-specific cut-off values for SO do not yet exist for this population, so the MRI findings reported here are best interpreted as complementary to, rather than independently diagnostic alongside, the DXA-based indices. Future work will implement spectroscopy validation and improved segmentation techniques to address the imaging limitations noted above.

## Conclusion

Given the small sample size of this interim cohort, these findings should be regarded as preliminary and hypothesis-generating rather than confirmatory. In this cohort of women undergoing laparoscopic sleeve gastrectomy, dual-modality body-composition assessment suggested that diagnostic index selection, more than imaging modality, was associated with whether sarcopenic obesity risk was detected: the standard ASM and ASM/height^2^ indices identified no patient at risk at any timepoint, while the weight-adjusted ASM/weight index identified ten of eleven patients at risk before surgery, decreasing to four of eleven by 6 months. This occurred alongside an absolute decrease in lean body mass and a parallel improvement in the relative muscle-to-fat ratio, observed with both DXA and MRI, consistent with a more favorable body-composition trajectory over this postoperative period even as absolute muscle mass declines; because this interim cohort pools participants from both arms of the parent trial, including a supervised exercise intervention that can itself influence muscle preservation, this trajectory should not yet be attributed to bariatric surgery alone. These findings are consistent with the rationale for weight-adjusted indices, as recommended by the 2022 ESPEN/EASO consensus statement, and raise the possibility that traditional height-adjusted indices may underestimate SO risk in patients with severe obesity; given the small, single-sex, single-ethnicity cohort studied here, we regard this as a hypothesis-generating observation rather than a basis for changing practice more broadly. Because functional muscle assessment was not available in this interim analysis, these results should be understood as risk stratification by body-composition criteria rather than a complete SO diagnosis; the full SarxOb trial, including functional testing, intervention-arm comparisons, and 18-month outcomes in a larger cohort, is expected to address this and the other limitations identified here.

## Data Availability

Individual, de-identified participant data supporting the conclusions of this article will be made available by the corresponding author upon reasonable request, in accordance with the ethics approval governing this trial.

## References

[B1] Batsis J. A. Villareal D. T. (2018). Sarcopenic obesity in older adults: aetiology, epidemiology and treatment strategies. Nat. Rev. Endocrinol. 14 (9), 513–537. 10.1038/s41574-018-0062-9 30065268 PMC6241236

[B2] Buzga M. Svagera Z. Tomaskova H. Hauptman K. Holeczy P. (2017). Metabolic effects of sleeve gastrectomy and laparoscopic greater curvature plication: an 18-month prospective, observational, open-label study. Obes. Surg. 27 (12), 3258–3266. 10.1007/s11695-017-2779-2 28674838

[B3] Buzga M. Pekar M. Uchytil J. Horká V. Malůš J. Vilímek D. (2023). Prevention of sarcopenia in patients with obesity after bariatric and metabolic surgery: the effect of programmed training on the muscle tissue and anthropometric functions - a randomized controlled trial (sarxOb study protocol). Biomol. Biomed. 23 (2), 191–197. 10.17305/bjbms.2022.7786 36154873 PMC10113941

[B4] Buzga M. Pekar M. Horka V. Hyvlova D. Jirik R. Uchytil J. (2025). Feasibility of implementing ESPEN/EASO consensus criteria for sarcopenic obesity assessment in bariatric surgery: a dual-modality imaging pilot study. Clin. Nutr. ESPEN 70, 650–659. 10.1016/j.clnesp.2025.11.009 41241217

[B5] Chaudry O. Friedberger A. Grimm A. Uder M. Nagel A. M. Kemmler W. (2021). Segmentation of the fascia lata and reproducible quantification of intermuscular adipose tissue (IMAT) of the thigh. MAGMA 34 (3), 367–376. 10.1007/s10334-020-00878-w 32761398 PMC8154773

[B6] Chen L. K. Lee W. J. Peng L. N. Liu L. K. Arai H. Akishita M. (2016). Recent advances in sarcopenia research in Asia: 2016 update from the Asian working group for sarcopenia. J. Am. Med. Dir. Assoc. 17 (8), 767. 10.1016/j.jamda.2016.05.016 27372539

[B7] Correa-de-Araujo R. Addison O. Miljkovic I. Goodpaster B. H. Bergman B. C. Clark R. V. (2020). Myosteatosis in the context of skeletal muscle function deficit: an interdisciplinary workshop at the national institute on aging. Front. Physiol. 11, 963. 10.3389/fphys.2020.00963 32903666 PMC7438777

[B8] Coutinho T. Goel K. Corrêa de Sá D. Carter R. E. Hodge D. O. Kragelund C. (2013). Combining body mass index with measures of central obesity in the assessment of mortality in subjects with coronary disease. J. Am. Coll. Cardiol. 61 (5), 553–560. 10.1016/j.jacc.2012.10.035 23369419

[B9] Crispim Carvalho N. N. Martins V. J. B. Filho J. M. de Arruda Neta A. d. C. P. Pimenta F. C. F. de Brito Alves J. L. (2023). Effects of preoperative sarcopenia-related parameters on the musculoskeletal and metabolic outcomes after bariatric surgery: a one-year longitudinal study in females. Sci. Rep. 13 (1), 13373. 10.1038/s41598-023-40681-w 37591922 PMC10435473

[B10] Cruz P. Johnson B. D. Karpinski S. C. Limoges K. A. Warren B. A. Olsen K. D. (2011). Validity of weight loss to estimate improvement in body composition in individuals attending a wellness center. Obesity 19 (11), 2274–2279. 10.1038/oby.2011.102 21566566 PMC4103167

[B11] Cruz-Jentoft A. J. Bahat G. Bauer J. Boirie Y. Bruyère O. Cederholm T. (2019). Sarcopenia: revised European consensus on definition and diagnosis. Age Ageing 48 (1), 16–31. 10.1093/ageing/afy169 30312372 PMC6322506

[B12] Donini L. M. Busetto L. Bischoff S. C. Cederholm T. Ballesteros-Pomar M. D. Batsis J. A. (2022). Definition and diagnostic criteria for sarcopenic obesity: ESPEN and EASO consensus statement. Obes. Facts 15 (3), 321–335. 10.1159/000521241 35196654 PMC9210010

[B13] Eisenberg D. Shikora S. A. Aarts E. Aminian A. Angrisani L. Cohen R. V. (2022). American society of metabolic and bariatric surgery (ASMBS) and international Federation for the surgery of obesity and metabolic disorders (IFSO) indications for metabolic and bariatric surgery. Obes. Surg. 33(1):3–14. 10.1007/s11695-022-06332-1 36336720 PMC9834364

[B14] Garneau L. Aguer C. (2019). Role of myokines in the development of skeletal muscle insulin resistance and related metabolic defects in type 2 diabetes. Diabetes Metab. 45 (6), 505–516. 10.1016/j.diabet.2019.02.006 30844447

[B15] Gomez-Ambrosi J. Andrada P. Valentí V. Rotellar F. Silva C. Catalán V. (2017). Dissociation of body mass index, excess weight loss and body fat percentage trajectories after 3 years of gastric bypass: relationship with metabolic outcomes. Int. J. Obes. 41 (9), 1379–1387. 10.1038/ijo.2017.134 28584299

[B16] Gonzalez Arnaiz E. Ariadel Cobo D. Estébanez B. Barajas Galindo D. Pintor de la Maza B. Urioste Fondo A. (2024). Prevalence of sarcopenic obesity according to different diagnostic methods and cut-off points in candidates for bariatric surgery. Clin. Nutr. 43 (5), 1087–1093. 10.1016/j.clnu.2024.03.015 38579371

[B17] Grimm A. Meyer H. Nickel M. D. Nittka M. Raithel E. Chaudry O. (2018). Repeatability of dixon magnetic resonance imaging and magnetic resonance spectroscopy for quantitative muscle fat assessments in the thigh. J. Cachexia Sarcopenia Muscle 9 (6), 1093–1100. 10.1002/jcsm.12343 30221479 PMC6240750

[B18] Grover B. T. Morell M. C. Kothari S. N. Borgert A. J. Kallies K. J. Baker M. T. (2019). Defining weight loss after bariatric surgery: a call for standardization. Obes. Surg. 29 (11), 3493–3499. 10.1007/s11695-019-04022-z 31256357

[B19] Haghighat N. Ashtary-Larky D. Bagheri R. Aghakhani L. Asbaghi O. Amini M. (2022). Preservation of fat-free mass in the first year after bariatric surgery: a systematic review and meta-analysis of 122 studies and 10,758 participants. Surg. Obes. Relat. Dis. 18 (7), 964–982. 10.1016/j.soard.2022.02.022 35581110

[B20] Heymsfield S. B. Gonzalez M. C. Lu J. Jia G. Zheng J. (2015). Skeletal muscle mass and quality: evolution of modern measurement concepts in the context of sarcopenia. Proc. Nutr. Soc. 74 (4), 355–366. 10.1017/S0029665115000129 25851205

[B21] Hoffmann C. Weigert C. (2017). Skeletal muscle as an endocrine organ: the role of myokines in exercise adaptations. Cold Spring Harb. Perspect. Med. 7 (11), a029793. 10.1101/cshperspect.a029793 28389517 PMC5666622

[B22] Hruska P. Kucera J. Pekar M. Holéczy P. Mazur M. Buzga M. (2022). Proteomic signatures of human visceral and subcutaneous adipocytes. J. Clin. Endocrinol. Metab. 107 (3), 755–775. 10.1210/clinem/dgab756 34669916 PMC8851937

[B23] Janochova K. Haluzik M. Buzga M. (2019). Visceral fat and insulin resistance - what we know? Biomed. Pap. 163 (1), 19–27. 10.5507/bp.2018.062 30398218

[B24] Ji T. Li Y. Ma L. (2022). Sarcopenic obesity: an emerging public health problem. Aging Dis. 13 (2), 379–388. 10.14336/AD.2021.1006 35371597 PMC8947824

[B25] Kim K. M. Jang H. C. Lim S. (2016). Differences among skeletal muscle mass indices derived from height-weight-and body mass index-adjusted models in assessing sarcopenia. Korean J. Intern Med. 31 (4), 643–650. 10.3904/kjim.2016.015 27334763 PMC4939509

[B26] Matos O. Ruthes E. M. P. Malinowski A. K. C. Lima A. L. Veiga M. S. Krause M. P. (2020). Changes in bone mass and body composition after bariatric surgery. Gynecol. Endocrinol. 36 (7), 578–581. 10.1080/09513590.2020.1762558 32406280

[B27] Molero J. Moize V. Flores L. De Hollanda A. Jimenez A. Vidal J. (2020). The impact of age on the prevalence of sarcopenic obesity in bariatric surgery candidates. Obes. Surg. 30 (6), 2158–2164. 10.1007/s11695-019-04198-4 32249368

[B28] Nuijten M. A. H. Eijsvogels T. M. H. Monpellier V. M. Janssen I. M. C. Hazebroek E. J. Hopman M. T. E. (2022). The magnitude and progress of lean body mass, fat-free mass, and skeletal muscle mass loss following bariatric surgery: a systematic review and meta-analysis. Obes. Rev. 23 (1), e13370. 10.1111/obr.13370 34664391 PMC9285034

[B29] O'Brien P. E. Hindle A. Brennan L. Skinner S. Burton P. Smith A. (2019). Long-term outcomes after bariatric surgery: a systematic review and meta-analysis of weight loss at 10 or more years for all bariatric procedures and a single-centre review of 20-Year outcomes after adjustable gastric banding. Obes. Surg. 29 (1), 3–14. 10.1007/s11695-018-3525-0 30293134 PMC6320354

[B30] Pietrobelli A. Wang Z. Formica C. Heymsfield S. B. (1998). Dual-energy X-ray absorptiometry: fat estimation errors due to variation in soft tissue hydration. Am. J. Physiol. 274 (5), E808–E816. 10.1152/ajpendo.1998.274.5.E808 9612238

[B31] Poggiogalle E. Lubrano C. Sergi G. Coin A. Gnessi L. Mariani S. (2016). Sarcopenic obesity and metabolic syndrome in adult Caucasian subjects. J. Nutr. Health Aging 20 (9), 958–963. 10.1007/s12603-015-0638-1 27791227 PMC12878523

[B32] Powell-Wiley T. M. Poirier P. Burke L. E. Després J. P. Gordon-Larsen P. Lavie C. J. (2021). Obesity and cardiovascular disease: a scientific statement from the American heart association. Circulation 143 (21), e984–e1010. 10.1161/CIR.0000000000000973 33882682 PMC8493650

[B33] Prado C. M. M. Wells J. C. K. Smith S. R. Stephan B. C. M. Siervo M. (2012). Sarcopenic obesity: a critical appraisal of the current evidence. Clin. Nutr. 31 (5), 583–601. 10.1016/j.clnu.2012.06.010 22809635

[B34] Rodrigues P. S. Mendonça F. M. Neves J. S. Luís C. Rodrigues I. Moreno T. (2024). Effects of bariatric surgery on sarcopenic obesity outcomes: a one-year prospective study in middle-aged women. Obes. Surg. 34 (5), 1674–1683. 10.1007/s11695-024-07164-x 38523172

[B35] Rosenberg I. H. (1997). Sarcopenia: origins and clinical relevance. J. Nutr. 127 (5), 990S–991S. 10.1093/jn/127.5.990S 9164280

[B36] Ruthes E. M. P. Lenardt B. C. C. Lass A. D. Petroski C. A. de Mello M. F. de Andrade Junior A. B. (2022). Lean mass and strength profile of women submitted to bariatric surgery: comparison of the EWGSOP2 and FNIH classification for sarcopenia - ASBS program phase II. Gynecol. Endocrinol. 38 (10), 868–873. 10.1080/09513590.2022.2119956 36067795

[B37] Schauer P. R. Bhatt D. L. Kirwan J. P. Wolski K. Aminian A. Brethauer S. A. (2017). Bariatric surgery *versus* intensive medical therapy for diabetes - 5-year outcomes. N. Engl. J. Med. 376 (7), 641–651. 10.1056/NEJMoa1600869 28199805 PMC5451258

[B38] Schautz B. Later W. Heller M. Muller M. J. Bosy-Westphal A. (2012). Total and regional relationship between lean and fat mass with increasing adiposity - impact for the diagnosis of sarcopenic obesity. Eur. J. Clin. Nutr. 66 (12), 1356–1361. 10.1038/ejcn.2012.138 23031852

[B39] Seabolt L. A. Welch E. B. Silver H. J. (2015). Imaging methods for analyzing body composition in human obesity and cardiometabolic disease. Ann. N. Y. Acad. Sci. 1353, 41–59. 10.1111/nyas.12842 26250623

[B40] Seo E. Kwon Y. Alromi A. Eledreesi M. Park S. (2024). A multifaceted and inclusive methodology for the detection of sarcopenia in patients undergoing bariatric surgery: an in-depth analysis of current evidence. Rev. Endocr. Metab. Disord. 25 (4), 677–689. 10.1007/s11154-023-09864-8 38427134

[B41] Sivakumar J. Chen Q. Sutherland T. R. Read M. Ward S. Chong L. (2022). Body composition differences between excess weight loss >= 50% and < 50% at 12 months following bariatric surgery. Obes. Surg. 32 (8), 2556–2566. 10.1007/s11695-022-06128-3 35648364 PMC9156838

[B42] Sizoo D. de Heide L. J. M. Emous M. van Zutphen T. Navis G. van Beek A. P. (2021). Measuring muscle mass and strength in obesity: a review of various methods. Obes. Surg. 31 (1), 384–393. 10.1007/s11695-020-05082-2 33159294 PMC7808984

[B43] Sjostrom L. Lindroos A. K. Peltonen M. Torgerson J. Bouchard C. Carlsson B. (2004). Lifestyle, diabetes, and cardiovascular risk factors 10 years after bariatric surgery. N. Engl. J. Med. 351 (26), 2683–2693. 10.1056/NEJMoa035622 15616203

[B44] Tabesh M. R. Abolhasani M. Zali M. R. Bagheri R. Alipour M. Cheraghloo N. (2022). The impact of bariatric surgery procedures on the modulation of cardiometabolic risk factors in patients with severe obesity: a 12-month follow-up. J. Int. Med. Res. 50 (10), 030006052211196. 10.1177/03000605221119657 36314880 PMC9629577

[B45] Tagliafico A. S. Bignotti B. Torri L. Rossi F. (2022). Sarcopenia: how to measure, when and why. Radiol. Med. 127 (3), 228–237. 10.1007/s11547-022-01450-3 35041137 PMC8960583

[B46] Van De Laar A. W. Van Rijswijk A. S. Kakar H. Bruin S. C. (2018). Sensitivity and specificity of 50% excess weight loss (50%EWL) and twelve other bariatric criteria for weight loss success. Obes. Surg. 28 (8), 2297–2304. 10.1007/s11695-018-3173-4 29484610

[B47] Vieira F. T. Godziuk K. Lamarca F. Melendez-Araujo M. S. Lima R. M. Prado C. M. (2022). Sarcopenic obesity diagnosis by different criteria mid-to long-term post-bariatric surgery. Clin. Nutr. 41 (9), 1932–1941. 10.1016/j.clnu.2022.07.006 35947895

[B48] Walowski C. O. Braun W. Maisch M. J. Jensen B. Peine S. Norman K. (2020). Reference values for skeletal muscle mass - current concepts and methodological considerations. Nutrients 12 (3), 755. 10.3390/nu12030755 32178373 PMC7146130

[B49] Wang Z. Deurenberg P. Wang W. Pietrobelli A. Baumgartner R. N. Heymsfield S. B. (1999). Hydration of fat-free body mass: review and critique of a classic body-composition constant. Am. J. Clin. Nutr. 69 (5), 833–841. 10.1093/ajcn/69.5.833 10232621

[B50] Yang Y. X. Chong M. S. Tay L. Yew S. Yeo A. Tan C. H. (2016). Automated assessment of thigh composition using machine learning for dixon magnetic resonance images. Magn. Reson Mater Phy 29 (5), 723–731. 10.1007/s10334-016-0547-2 27026244

